# Prevalence, Caries, Dental Anxiety and Quality of Life in Children with MIH in Brussels, Belgium

**DOI:** 10.3390/jcm11113065

**Published:** 2022-05-29

**Authors:** Tania Vanhée, Julie Poncelet, Shereen Cheikh-Ali, Peter Bottenberg

**Affiliations:** Oral Health School, Université Libre de Bruxelles, 1070 Brussels, Belgium; julie.poncelet@ulb.ac.be (J.P.); shereen.cheikh-ali@ulb.ac.be (S.C.-A.); peter.bottenberg@ulb.be (P.B.)

**Keywords:** molar incisor hypomineralization, dental anxiety, quality of life

## Abstract

Molar incisor hypomineralisation (MIH) is a dental enamel pathology responsible for unfavorable functional and aesthetic implications. The objective of this study is to assess the prevalence, dental anxiety, and quality of life related to oral health in children with MIH. In 14 schools in Brussels, Belgium, 290 children aged 8 to 9.5 answered Children’s Fear Survey Schedule-Dental Subscale (CFSS-DS) and Child-Oral Impact on Daily Performance (C-OIDP) questionnaires to assess dental anxiety and quality of life related to oral health (OHRQoL). Oral examinations allowed us to detect MIH according to standardized criteria. The MIH prevalence was 18.6%. The Decayed, Missing and Filled Teeth index (DMFT index) of MIH patients was significantly higher than non-MIH patients (*p* < 0.001), mainly due to more restored teeth. There was no significant association between MIH and dental anxiety or OHRQoL. Caries in the deciduous dentition was significantly associated with impaired quality of life. The MIH prevalence in Brussels is comparable to other European countries. MIH had no significant impact on dental anxiety and OHRQoL in this sample. The dynamic nature of MIH lesions requires early diagnosis and management to limit the evolution of the severity of the lesions and their implications. It is possible that older age groups may present more symptoms, however, this would require a longitudinal study.

## 1. Introduction

Molar-Incisor Hypomineralisation (MIH) is a developmental disturbance of dental enamel of unknown etiology [1]. As the name implies, it is found on molars and, to a lesser extent, incisors, as well in the primary as permanent dentition. Lesions are qualitative defects showing as delimitated whitish-opaque spots on the enamel of normal thickness, and are clearly visible after tooth emergence. With time, the lesions may acquire external stains. An increased enamel porosity makes the involved teeth more vulnerable to dental caries or fracture [2]. Furthermore, in more pronounced cases the porosity renders teeth more sensitive to stimuli during toothbrushing or chewing, thereby decreasing quality of life and posing an obstacle to oral hygiene [3].

According to Schwendicke et al., in 2018 the global prevalence of MIH had a mean (95% CI) of 13.1% (11.8–14.5%), with regional differences. An estimated prevalence of 15% for the Belgian population has been calculated based on extrapolation [4]. However, to date no data based on fieldwork are available.

Next to data on prevalence alone, data on a possible association between the prevalence of MIH and oral health-related quality of life, dental anxiety, and caries experience are unconclusive. Several authors have stated that frequent (re-)restoration due to MIH might increase dental anxiety in children and the associated painful experience might decrease their quality of life [5,6]. However, this could not be confirmed in other cohorts.

Teeth with MIH are more sensitive than healthy teeth for daily oral hygiene care and during their therapeutic management. It makes sense, then, to wonder what the oral health-related quality of life and dental anxiety in patients with MIH might be compared to patients without MIH.

Therefore, the research questions of this study were: (1) What is the prevalence and severity of MIH in a group of 8-year-olds from schools in Brussels, Belgium? (2) Is there an association between MIH and dental caries experience? (3) Is there a difference in oral health-related quality of life and dental anxiety between children affected by MIH or those not affected?

The first null hypothesis is therefore that MIH and dental anxiety are independent. The second null hypothesis is that MIH and oral health-related quality of life are independent.

## 2. Materials and Methods

### 2.1. Ethical Review

The study protocol obtained a positive ethical review from the Ethics Committee of the Saint-Pierre University Hospital on the 9 December 2019 (protocol code B076201942430). The guardians of the children gave written informed consent, and permission was obtained by the school health board prior to the examination.

### 2.2. Sample Selection and Size

As early detection of MIH is advocated at the age of 8 years, sampling took place in the third year of primary school in Brussels [7]. We aimed for a sample size of 300 [8]. 

Inclusion criteria were: being between 8 years and 9 ½ years of age, presenting no fixed orthodontic appliances, and having a valid signed consent form at the day of the examination. Children presenting with discolorations or hypoplasias other than MIH were excluded (fluorosis, amelogenesis imperfecta, tetracyclin stain). From the 28 schools contacted, 15 provided authorization for the examination to be performed in their premises, resulting in 327 children being present at the day of the examination. A ranking published in 2019 by the Government of the French Community estimates the average socio-economic level of enrolled students by school and assigns a score from 1 to 20 per establishment. All the schools included in the study had a low or moderate socio-economic index, between 1 (very low) and 13 (intermediate) [9].

### 2.3. Clinical Examination

Two trained and calibrated observers performed examinations and recordings. Training was performed using a series of photographs according to the method described by Ghanim et al. in 2017, and resulted in a Kendall’s Tau correlation coefficient ranging from 0.539 to 0.722 (*p* < 0.001) with raters to reference standard as well as inter-rater [10].

Data collection took place after an introductory presentation to the class. Clinical examination took place in a separate room using an LED head lamp and a disposable mirror after cleaning and drying teeth with a gauze swab. All present teeth were recorded. MIH was scored according to the method described by Ghanim et al. in 2015 [11]. A categorical index was used to describe the prevalence of MIH, with 0 = no signs of pathology, 1 = signs of pathology not related to MIH, 2 = limited opacity, 3 = posteruptive enamel fracture, 4 = atypical restoration, 5 = atypical carious lesion 6 = extracted due to MIH., and 7 = missing data, unable to obtain a score. Next to prevalence, extension of the lesion was recorded as 0 = unaffected < 1/3, 2 = between 1/3 and 2/3 and 3 > 2/3 of the crown. Dental caries was registered according to the WHO criteria at the level of dentinal involvement (D3). Sequels of caries or other pathologies were registered according the PUFA index [12]. 

Data about age and sex as well as previous dental visits were registered. For further data processing, certain data were aggregated or dichotomized (see further statistical analysis). A written report was handed over in a closed envelop to parents/guardians via the teaching staff.

### 2.4. Questionnaires

The questionnaires used in this study aimed to assess dental fear and oral health-related quality of life using the French translations of validated instruments [13,14].

Questions were asked orally and answers noted on a paper document. Data were later transferred to a spreadsheet.

Dental fear was assessed using Children’s Fear Survey Schedule-Dental Subscale (CFSS-DS), resulting in data categorized on a 5-point Likert scale ranging from 1 to 5. The questionnaire contained 15 items related to dental or medical environments (sensory stimuli, instruments and interventions) [15].

The questionnaire was distributed in the classroom and questions were read aloud by the examiners. For further data processing, the mean plus 1 SD (38) was used as a cut-off between no or manageable fear and fear compromising a medical treatment.

Furthermore, the Child-Oral Impact on Daily Performance (C-OIDP) index was used. It contained eight items with six categorical answers (0, never, to 5, nearly every day). Questions were asked individually following the clinical examination [14].

Following a pilot test run, frequency was noted as the only scoring intensity proven to be time-consuming, and led to unreliable results. For further data processing, data were dichotomized into “no impact” (0) vs. “any impact” (>0).

### 2.5. Data Processing

After data entry, files were checked and entered in an SPSS (Armonk, NY, USA) database for further analysis. Descriptive data were obtained. Further analysis was performed on aggregated or dichotomized data. Caries experience was transformed in the sum of decayed and filled teeth (DF) for the permanent dentition and DEF (decayed, extracted, filled) for the deciduous dentition.

A binary stepwise logistic regression was performed with impact on quality of life (no vs. any) and fear of dental treatment (below vs. above cut-off value) as dependent variables. Associations were calculated with the independent variables MHI prevalence (Y/N) and severity (limited vs. extensive lesions), caries in deciduous and permanent dentition (number of decayed and filled teeth), dental visit (Y/N), school deprivation (1–13), age (by group), and sex, using the Wald criterion for significance and resulting in a beta coefficient for relative risk. Further explorative analyses were performed using non-parametric tests for more detailed aspects, such as MIH severity and esthetic impact.

## 3. Results

Of the 327 children, 37 were excluded because of age or the presence of fixed orthodontic appliances. Data from 290 children were available for analysis, consisting of 151 girls and 139 boys, mean age 8.4 y +/− 0.4. There was no inhomogeneous male/female distribution regarding age groups (X2, *p* = 0.21), and 91% of children said they had already had at least one dental visit in their life.

290 children were included in the sample; however, one subject was excluded for lack of data (n All Children = 289). Regarding the analysis of patients with or without MIH signs, ten other subjects were excluded because they had incompletely erupted first permanent molars (n Signs of MIH= 54, n No signs of MIH = 225). (Table 1)

**Figure 1:**
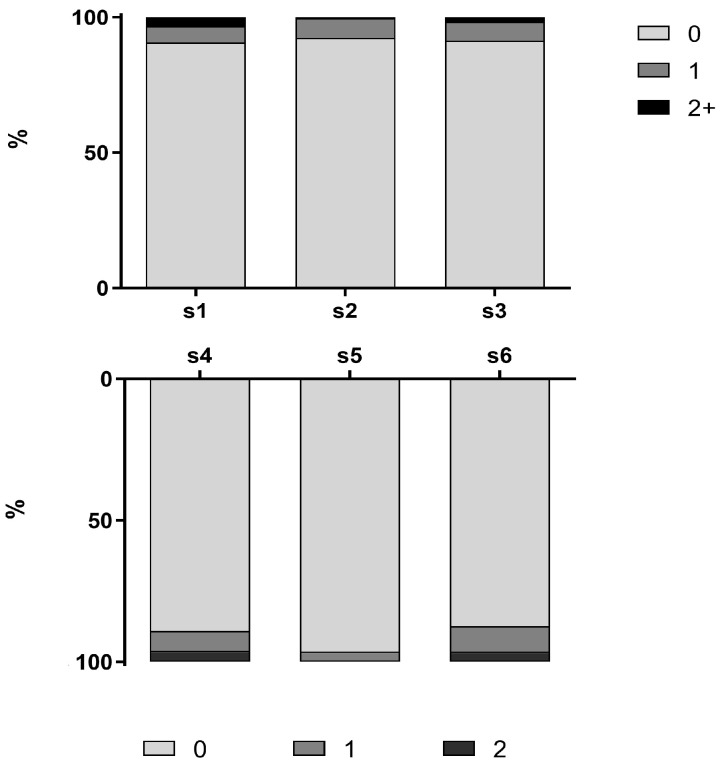
Distribution of MIH-affected teeth per sextant and the extension of the lesions (0: no signs of MIH, 1: small lesions, 2+ lesions encompassing several surfaces).

In 54 children, at least one tooth showed signs of MIH (18.6%, Figure 1), with a median number of three affected teeth (range 1 to 8). Of these, 18 children showed limited (extension score 1) and 36 showed extensive lesions (extension score 2 and 3). In the severe cases, a higher number of affected teeth could be observed (median 4, range 1–8) than in the mild cases (median 1.5, range 1–5). This difference was significant (MW test, *p* < 0.001). Cases with higher extension of the lesions showed more frequently involvement of the upper incisors. In twelve cases, atypical restorations could be found ten of which were in dentition otherwise free of signs of decay. In all twelve cases, atypical restorations were combined with untreated enamel fractures (PEB) on other teeth.

There was no difference in proportion between girls and boys regarding presence or absence of MIH (X2, *p* = 0.222) or for severity (X2, *p* = 0.519). 

Nine children showed other types of enamel hypoplasia (score 1). In 27 children, hypoplasia in deciduous molars was detected, of which 13 showed signs of MIH in the permanent dentition. 

Caries experience was high in the deciduous dentition, with 30.8% being caries-free (Table 2). Children showing signs of decay had a median of two (range 0–8) affected deciduous teeth. In the permanent dentition, 43 children showed signs of dental decay, of which 36 had affected deciduous teeth. Children affected by MIH did not have more decayed permanent teeth than non-affected children (MW, *p* = 0.195) although they had a significantly higher number of filled teeth (MW, *p* < 0.01, Table 1).

Regarding impact on quality of life, 70% of children reported an impact (QoL > 0). The median cumulative index was two (range 0–29). The item most frequently mentioned was impaired eating (any impact = 37%), while the least frequently mentioned impact was on school performance (any impact = 9%). Logistic regression analysis revealed no significant association with MIH, however, there was an association with caries experience in the deciduous dentition (Table 2). The significantly associated variable was school deprivation index, with higher ranked (less deprived) schools showing a lower impact on quality of life. However, when a more detailed analysis was performed regarding impaired esthetics (are you reluctant to smile?), children with MIH-affected upper incisors more frequently reported an impairment (MW, *p* = 0.03).

Dental anxiety was present in 40 children (14%); the most anxiogenic items were fear of an injection (70% > 0) and suffocating (69% > 0), while the least anxiogenic was undergoing a dental prophylactic cleaning (20% > 0). Anxiety was not associated with caries experience or with the presence or extension of MIH. However, having had no previous dental visit was significantly associated with higher anxiety scores. Furthermore, girls more frequently reported dental anxiety.

## 4. Discussion

In clinical practice, dentists often observe difficulties in treating teeth with MIH lesions, in particular when these lesions are severe. Indeed, these teeth are known to present difficulties in responding adequately to local anesthesia, and therefore require adapted and progressive management both behaviorally and technically [16]. Considering the state of anxiety exhibited by certain patients with these lesions, one would expect a correlation between dental anxiety and the severity of MIH. Our study is not in agreement with this clinical feeling; there was no association between the presence or severity of MIH and quality of life or dental anxiety.

However, the presence of MIH was associated with more restored permanent teeth. Caries prevalence in the deciduous dentition was high, and was associated with reduced quality of life.

The strong point of this study is that data for at least a part of the Belgian population are now available. The present study showed that in a convenience sample of school children in Brussels, the prevalence of MIH was 18%. These data suggest that the prevalence is higher than in the estimates made by Schwendicke based on a mathematical model (13.1%) [4].

The weakness of this study lies in the fact that the data are cross-sectional and only pertain to Brussels, a population characterized by a fairly heterogeneous socio-cultural background. A possible selection bias could have been the low socio-economic level of the school neighborhood. However, data collection was interrupted by the outbreak of the COVID-19 pandemic and could not be resumed at present.

In addition to the prevalence of MIH, dental caries and restorative status was assessed. Children with MIH had a significantly higher number of restored permanent teeth, most likely due to repair of defects caused by MIH, as shown in a previous review [17] and cross-sectional study [2]. The impact on quality of life was not significant in this survey, with the exception of esthetic concerns in children presenting affected incisors. Socio-economic factors as expressed by the school deprivation index played a role, as reported by Portella, although the variables in that study were chosen in a different way according to the circumstances [5]. Guttiérez found a significant association between QoL and MIH, however, their sample had a much higher prevalence of dental caries [18]. It is conceivable, considering the high prevalence of MIH in our sample, that children may develop symptoms later in the course of their life, as lesions tend to progress. Management of lesions is not straightforward and restoration margins tend to be unstable unless rather invasive procedures are chosen. However, at the age of the examination this could not yet be observed. The only answer to this would be a prospective study that is, considering the unpredictable restrictions due to the COVID-19 pandemic, very difficult to perform at the moment.

Dental fear may be associated with the presence of MIH, probably due to more frequent interventions. The data relative to this association are conflicting and depend to an extent on the design of the study, such as age group, presence of other pathologies, and study design. Parent-reported anxiety was higher in a group of Brazilian schoolchildren who had experience of dental treatment, while the association was reversed in our study [19]. It is possible that the availability of specialized child dental care in several university centers and private practices in the Brussels region might explain the difference. Kosma et al. have previously showed a higher anxiety score in girls, as in the present study [2].

Our results concerning quality of life did not show any differences between children with MIH and those without MIH. These results are not in agreement with those of the study by Joshi et al., which showed a difference between these same two groups in which there was a negative impact of MIH on quality of life. However, the cohorts studied are not comparable, as the study by Joshi et al. was carried out in a pediatric dental clinic while our study was carried out in a more neutral environment in terms of patient expectations, namely, schools [20]. In 2021, Michaelis et al. came to the same conclusion by comparing a cohort with caries without MIH and another with MIH. If the two pathologies had a negative effect on quality of life, patients with MIH showed a greater deficiency at this level. As in the study by Joshi et al., the subjects studied came from a pediatric dentistry consultation presenting this same selection bias [21]. A systematic review of the recent literature described this negative impact on quality of life, however, the studies included were all carried out in consultation. On the other hand, this review shows that MIH has no impact on dental anxiety, which is in agreement with our results [22]. Be that as it may, one of the strengths of our study is the removal of this selection bias.

## 5. Conclusions

In this study, no association could be found between MIH and dental anxiety. Impact on quality of life was limited to cases with lesions in the upper incisors. However, prevalence was high enough to be of concern to oral health specialists, as symptoms may develop in the course of these patients’ lives. This should be subject of prospective studies.

## Figures and Tables

**Table 1:** Outcomes related to dental caries experience in the whole sample and specific for children showing signs of MIH vs. not showing signs. Data in bold and marked with * are significantly different between children with or without signs of MIH.

Dental Caries Experience Indicators	All Children (*n* = 289)	Signs of MIH (*n* = 54)	No signs of MIH (*n* = 225)
DMFT, median (range)	**0 (0–5)**	**0 (0–4)**	0 (0–4) *
dmft, median (range)	2 (0–11)	2 (0–8)	2 (0–11)
Presence of decayed permanent teeth (*n*, %)	21 (7%)	6 (11%)	14 (6%)
Presence of decayed deciduous teeth (*n*, %)	115 (40%)	23 (43%)	86 (38%)
Presence of filled permanent teeth (*n*, %)	**26 (9%)**	**11 (20%)**	13 (6%) *
Presence of filled deciduous teeth (*n*, %)	152 (53%)	27 (50%)	118 (52%)
Presence of missing permanent teeth (*n*, %)	0	0	0
Presence of missing deciduous teeth (*n*, %)	52 (18%)	10 (18%)	41 (18%)

**Table 2:** Results of the binary logistic regression on variables associated with impact on quality of life and dental anxiety. The significance (*p* value) according to the Wald test (Wald) is provided, as is the relative risk (Exp(B)). Values in bold are significant.

	Impact on Quality of Life (Y/N)			Dental Anxiety (Y/N)		
**Variable**	**Wald**	**Exp(B)**	**Sig.**	**Wald**	**Exp(B)**	**Sig.**
**MIH prevalence**	0.264	1.317	0.607	0.026	0.718	0.873
**MIH severity**	0.001	0.262	0.976	1.125	1.017	0.289
**Nr of MIH affected permanent teeth**	0.557	1.565	0.456	1.808	1.183	0.179
**Nr of MIH affected primary teeth**	3.183	1.055	0.074	0.021	0.627	0.884
**Decayed and filled permanent teeth**	0.013	1.208	0.909	0.237	1.027	0.626
**Decayed and filled deciduous teeth**	**3.974**	**0.918**	**0.046**	1.069	1.136	0.301
**School deprivation**	**8.686**	**1.059**	**0.003**	1.095	0.898	0.295
**Dental visit (Y/N)**	0.628	0.257	0.428	**7.071**	**1.449**	**0.008**
**Age group**	2.218	1.414	0.136	1.981	1.334	0.159
**Sex**	1.867	3.547	0.172	**9.728**	**0.683**	**0** **.** **002**

## Data Availability

Data available in a publicly accessible repository. The data presented in this study are openly available at this link: https://figshare.com/articles/dataset/Results_MIH_doc/19603600 with the DOI:10.6084/m9.figshare.19603600.
